# Influence of Pre-Exposure on the Material Response of Epoxy-Based Volume Holographic Recording Material

**DOI:** 10.3390/polym14112193

**Published:** 2022-05-28

**Authors:** Tina Sabel-Grau

**Affiliations:** Department of Chemistry, Technische Universität Berlin, Strasse des 17. Juni 115, 10623 Berlin, Germany; tina@physik.tu-berlin.de

**Keywords:** photosensitive polymers, volume holographic gratings, diffractive optical elements, material response, pre-exposure, holographic grating formation, refractive index contrast

## Abstract

The formation of volume holograms in photosensitive polymers is a complex process under the influence of many interacting factors: material composition and processing, exposure conditions, and pre-exposure affect the development and final characteristics of holographic gratings. In order to better understand the interplay of these influencing factors, the detailed investigations of holographic recording in a new organic material are performed and the results are presented here. The material response and performance of an epoxy-based free surface material designed for volume holography are investigated. For this purpose, time-resolved investigation of volume holographic grating growth is performed on the one hand. Spatially resolved analysis of volume holographic phase gratings by point-by-point scanning of the local material response to the Gaussian intensity distribution of the recording beams is carried out on the other hand. Thus, the influence of pre-exposure on the temporal grating formation, as well as on the final obtained refractive index contrast, was determined. The various effects observed can be explained by the consumption of photosensitive compounds and prior crosslinking in the course of pre-exposure. Rather unexpected effects are that, on the one hand, pre-exposed gratings emerge with ever more complete null diffraction at the transition point and, on the other hand, a stabilizing effect of some degree of pre-exposure on regions exposed with low intensity was identified.

## 1. Introduction

In the research of volume holographic materials for diverse applications, much emphasis is placed on their design and understanding of the grating formation processes [1]. The versatile applications are numerous and range from recording media, holographic data storage, self-written waveguides, and wavelength-selective devices, to solar energy concentrators and diffractive elements for biomedical applications [2,3,4,5,6,7]. Therefore, new materials for volume holography are constantly being developed and these are being researched in ever greater depth [4,8].

As a volume holographic material, photosensitive polymers represent a particularly interesting group among stimuli-responsive polymeric materials, standing out due to their ability to be applied in a non-invasive and easily controlled manner [9]. Light as a stimuli entails optical structuring by the application of volume holography as a single-step method for manufacturing 3D diffractive micro- and nanostructures [10].

The mechanism of volume hologram formation in photosensitive polymers, a complex process where several components are involved, is ruled by the interplay of polymerization and diffusion, induced by a spatially modulated holographic exposure [3,11]. Studying the light-induced material response allows conclusions to be drawn on the mechanism of volume hologram formation [12,13]. Hereby, many factors influence how a photosensitive material responds to light during a holographic exposure. In general, intrinsic material parameters, such as material composition or viscosity, can be distinguished from extrinsic factors, i.e., recording parameters, such as exposure duration and recording intensity, as well as pre-exposure. However, the influence of pre-exposure has not yet been sufficiently investigated.

The material response is studied to predict the behavior of the materials. Diffusion models are then used for this purpose [14,15]. This also includes studying the dynamics of noise gratings with the goal of developing material formulations and processing methods that result in high signal-to-noise ratios [16]. It should be noted that the individual factors can also influence each other [17]. For example, a curing process may allow stabilization of the hologram, but at the same time may lead to a decrease in diffraction efficiency [18].

In the exploration and optimization of new materials for volume holography, the determination of the material response to the exposure conditions is of paramount importance, since otherwise—i.e., with insufficient knowledge—it will overlap with the material response in the study of the diffraction properties. In other words, if we do not know the effects of each factor, we do not know whether the change in material composition or processing on the one hand, or the exposure or pre-exposure conditions on the other, affected the material response. Mostly, the material response to energy density of exposure—the product of exposure intensity and exposure duration—is studied and well understood, while the influence of pre-exposure is mostly neglected. However, it immediately plays a role as soon as more than one hologram is generated per sample. In fact, the pre-exposure level accumulates by means of scattered light from grating to grating. With a correspondingly high sensitivity of the material—which is certainly desirable—previously generated holograms influence the formation of subsequently generated holograms.

Beyond the rather disturbing superimposed influence of previous exposures, considering incoherent pre-exposure as an integral part of a holograph’s toolkit for perfecting imaging results for volume holographic elements is also proposed [19]. In order to be able to do this, however, it is first necessary to know the influence of pre-exposure precisely, so that incoherent exposure can then be dosed and used in a targeted manner.

A straightforward option to study the influence of such pre-exposure on grating formation and material performance is to generate several holograms—under identical exposure conditions—one after the other on a sample, observe their formation in situ, and then evaluate the individual diffraction characteristics of each hologram.

In the first place it is necessary to estimate to what extent and in what way pre-exposure of a particular sample may influence grating formation of subsequently generated holograms. In particular, when observing grating formation for several minutes up to hours, holograms are generated in one sample with appropriate time delay. In this case, disregard of the pre-exposure by means of scattered light of preliminary exposures may significantly distort interpretation of results. Sizable long exposure durations of several seconds further reinforce this effect.

This work is intended to help clarify the influence of pre-exposure. The investigations presented here are based on a novel, recently introduced, epoxy-based material for volume holography, standing out due to its high diffraction efficiency and dimensional stability, low shrinkage, and great resistance to environmental conditions, having the special feature of a free surface [20].

## 2. Materials and Methods

### 2.1. Sample Preparation

**Figure 1:**
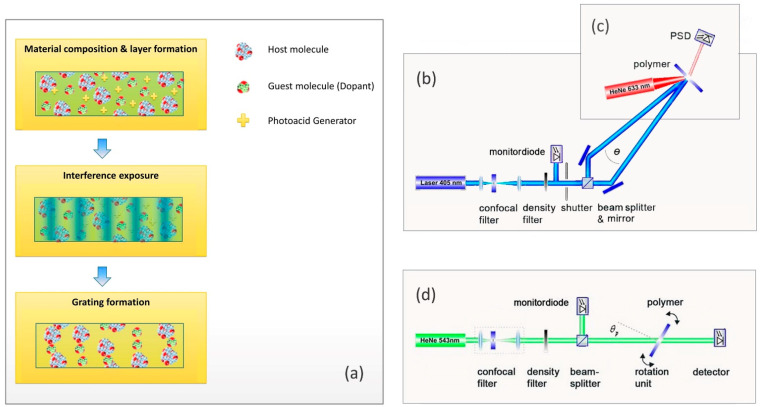
Overview on Materials and Methods: (**a**) Schematic illustration of composition, exposure, and grating formation for the host–guest material. (**b**) Holographic exposure setup with geometry for transmission gratings. (**c**) In situ setup for the investigation of hologram formation in real time. (**d**) Rotation-scan setup for angular resolved analysis of final gratings.

Free surface, ultraviolet curable epoxy-based samples were prepared by micro resist technology GmbH (Berlin, Germany). Both host and guest molecules featured epoxy functional groups with the corresponding mechanism of cationic ring-opening polymerization. The refractive indices of the host and guest components at 589 nm were *n*_host_ ≈ 1.58 and *n*_guest_ ≈ 1.46, respectively. A sensitized photoacid generator (PAG) was used to induce cross-linking by cationic polymerization at 405 nm. A schematic illustration of the host–guest material composition and grating formation mechanism is shown in Figure 1a.

Spin coating of the material in a solution on glass substrates with a rotation speed of 800 min^−1^ resulted in a layer thickness of 200 µm. A subsequent pre-exposure bake was carried out on a hotplate (80 °C) for 30 min to drive out the remaining solvent in order to obtain a tack-free film. For more details on the host–guest system, in terms of composition as well as performance—such as energetic sensitivity and angular selectivity—see [20].

### 2.2. Holographic Exposure

All investigations are based on one-dimensional, plane-wave, transmission type volume holographic gratings. Symmetric recording geometry was applied to create non-slanted gratings, with a periodicity of Λ ≈ 3 µm. Holographic exposure was performed by two freely propagating, s-polarized recording beams with a wavelength of *λ*_exp_ = 405 nm, a beam diameter of 2 mm, and laser power of (0.7 ± 0.3) mW per beam. A schematic illustration of the holographic exposure setup is shown in Figure 1b.

After the completion of holographic grating formation, samples were fixed by a UV flood cure with a dose of 350 mJ/cm^2^. During this curing step the remaining photoinitiator is used up, resulting in a sample which is no longer light-sensitive. No postbake, hardbake, or any additional developing was applied.

### 2.3. Hologram Characterization

#### 2.3.1. Real-Time Observation of Holographic Grating Growth

Grating growth curves are obtained by monitoring the time evolution of the diffracted part of a probe beam from the very start of exposure. Such in situ techniques enable real-time, non-disturbing observation of the grating formation process [2,21].

To ensure non-disturbing observation, the in situ probe wavelength was chosen outside of the absorption spectrum of the photosensitizer dye. A fiber-guided 633 nm HeNe laser was used in combination with an adjustable collimator. This allows probing with a slightly focused beam to steadily ensure a stable on-Bragg condition. A position sensitive device (PSD) was used to detect the diffracted light. The PSD provides time-resolved information on the diffraction efficiency. A schematic illustration of the in situ setup for real-time observation of holographic grating growth is shown in Figure 1c.

#### 2.3.2. Angular-Resolved Analysis and Lateral Scanning

Angular-resolved investigations allow the determination of the Bragg selectivity, which defines the optical functionality [22]. Analysis of the final holograms was accomplished in a rotation-scan setup with a collimated probe beam. The rotation-scan setup is shown in Figure 1d. The transmitted signal of a HeNe laser (*λ*_p_ = 543 nm) is detected while the hologram under test is rotated. The diffraction efficiency was calculated from the angular resolved transmission. By comparison of the angular resolved diffraction efficiency with a rigorous solution of the coupled wave theory (RCWT) [23], the layer thickness d and the refractive index contrast Δ*n* is derived.

The probe beam featured a diameter of 0.2 mm. Probing less than a tenth of the exposed area was primarily for the purpose of measuring precision [20,24]. However, it also enables the scanning of the grating by moving the sample perpendicular to the optical axis. A sequence of rotation scans through the grating diameter constitutes a lateral scan [25]. This analytical method allows the determination of the hologram characteristics along the sample surface. Thereupon, it is possible to compare and track respective properties from the center of the grating to the edges, corresponding to the areas of highest and lowest recording intensity. As a consequence, spatial sequences of the grating parameters are derived, providing insight into the local material characteristics. Furthermore, every single lateral position is assigned to a certain local exposure dose, determined by the Gaussian intensity distribution of the recording beams. As a consequence, a lateral scan contains energetically resolved information as well. The respective allocation of the abscissa to the local energy density allows one to draw conclusions on the influence of the recording dose.

## 3. Results and Discussion

### 3.1. Real-Time Observation of Holographic Grating Growth

Holographic grating growth in the novel epoxy-based material system has been studied extensively and can be described in general as a two-step growth, ruled by the interplay of polymerization and diffusion [21]. It can be described by the Δ*n*-transition theory, according to which the first step of growth is attributed to a positive refractive index contrast Δ*n*_+_, while the second growth step is related to segregation of components by diffusion, resulting in a permanent grating with negative refractive index contrast Δ*n*_−_. Overall results for the temporal evolution of refractive index contrast:

$$\Delta n\left(t\right)=\Delta n_{+}\left(r_{+},s_{+},t_{0+},t\right)+\Delta n_{-}\left(r_{-},s_{-},t_{0-},t\right)$$

While the individual growth of the positive contrast grating is ruled by $\Delta n_{+}\left(t\right)=s_{+}\left[1-e^{-r_{+}\left(t-t_{0+}\right)}\right]$, hereby *r*_+_ controls the rise, *s*_+_ is the saturation value, and *t*_0+_ is the starting time of grating growth. The same applies for the negative contrast grating with Δ*n*_−_. The change of sign in Δ*n*(*t*) causes a zero point of diffraction—the transition point at the transition time *t* = *t*_T_; here the grating growth curve passes through its minimum. For the total resulting permanent grating, the saturation value is $s=s_{+}+s_{-}=\Delta n\left(t\overset{}{\to }\infty \right)$.

#### 3.1.1. The Doped System

**Figure 2:**
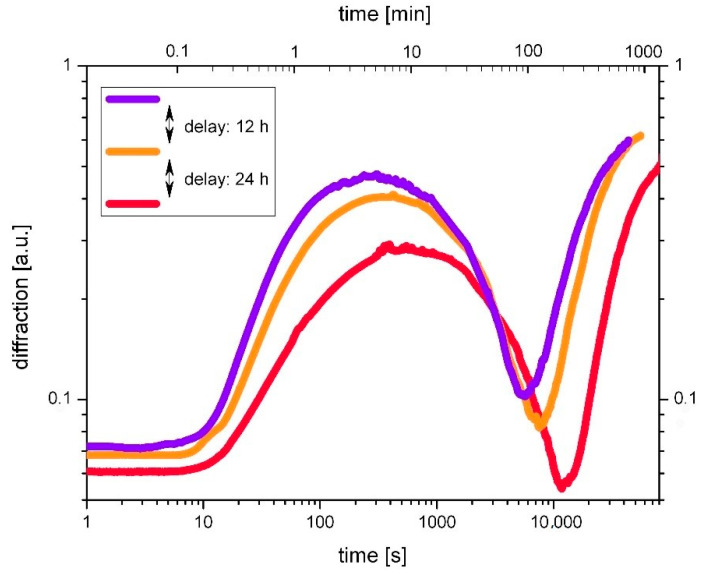
Impact of pre-exposure on the holographic grating growth in the doped system. Holograms were subsequently recorded in one sample with a time lag of 12 h. Exposure duration was 15 s for each hologram and the corresponding exposure dose was 750 mJ/cm^2^. The growth curves are displayed on double logarithmic scales to highlight the differences in the formation of the first (violet), second (orange), and third (red) grating.

Figure 2 shows growth curves for three holograms, subsequently recorded with a time lag of 12 h each. Exposure duration was 15 s for each hologram. From grating to grating the pre-exposure level accumulates by means of scattering. On the double logarithmic scale, the individual growth curves can easily be compared. Four types of differences can be identified:

(1) Lower rise of the first growth step, i.e., decrease of the parameter *r*_+_. The steepest rising positive-contrast-growth is achieved with the lowest pre-exposure dose. This effect can be traced back to the fact that photosensitive compounds are partially used up during exposure with the result of decreasing photosensitivity.
(2) Retarded transition point *t*_T._ Meeting the expectations out of the Δ*n*-transition theory, it is apparent from Figure 2 that from exposure to exposure, the transition point *t*_T_ is located at later times, indicating slower diffusion. This fact is plausible under the conception of a certain loss in mobility of the matrix throughout prior cross-linking, thereby impeding particle movement.
(3) Lower rise of the second growth step, i.e., decrease of the parameter r_−_: this can be apprehended as a consequence and co-action of the first two effects. As expected from the Δ*n*-transition theory, the rise of the second growth is progressively reduced with increased pre-exposure dose.
(4) Less incomplete null. The effect of an incomplete zero in the growth curve can be attributed to a non-zero average value of the diffraction efficiency due to significantly unequal transition times across the probe beam diameter, which could be the case if the polymerization and/or diffusion rates are intensity dependent. Here the influence of the pre-exposure is difficult to explain. Subsequently generated gratings show a less and less pronounced incomplete null.

Overall, the results lead to the conclusion that the more pre-exposure the sample undergoes, the less strongly the grating growth rises and the later diffusion starts and proceeds. However, the most conspicuous feature regards the completeness of the zero point throughout the transition. While the first growth curve shows a pronounced incomplete null, subsequent gratings emerge with ever more complete null diffraction at the transition point. Explanation of this effect remains outstanding.

#### 3.1.2. The Undoped System: SU-8

In studying the dynamics of the growth of holographic gratings, it has proven helpful to also investigate a simplified system in the form of the undoped SU-8 [12]. Without guest component the system is vastly simplified. The growth curves in case of SU-8 differ from those described by the Δ*n*-transition theory, since without guest component no transition takes place. However, they also show two phases, which can be explained by the formation of transient absorption as well as phase gratings in SU-8 [12].

**Figure 3:**
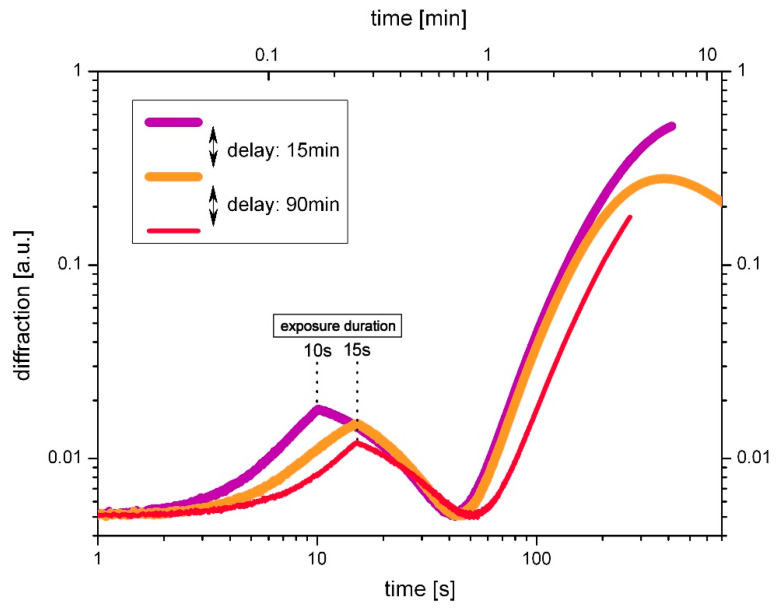
Impact of the pre-exposure on the grating growth in the undoped SU-8. Exposure duration was 10 s (violet curve) and 15 s (red and orange curve), respectively.

Figure 3 shows growth curves of three subsequently generated holograms with comparable exposure duration in the undoped system SU-8. Time delay was 15 min between the first and the second exposure and 90 min between the second and the third exposure. Again, results from Figure 3 illustrate that pre-exposure cannot be neglected.

As before with the doped system, the effect of pre-exposure on the grating growth affects all four characteristics of the growth curves:

(1) The initial growth of subsequent holograms rises slower, indicating a consumption of photoacid by previous exposures.
(2) The depletion area is slightly shifted to later times.
(3) The rise of the second growth step is decreasing with the number of pre-exposures. The two latter issues suggest deceleration of the cross-linking.
(4) Finally, the saturation and decrease of diffraction starts earlier in a pre-exposed sample (apparent in view of the orange curve in Figure 3). This meets the expectations with respect to the reduction of the dynamic range as a result of photoacid consumption and exhaustion of non-cross-linked chains.

### 3.2. Analysis of Final Gratings

#### 3.2.1. Impact of Pre-Exposure on the Intensity Response

To determine the influence of pre-exposure on the material response, the technique of lateral scanning was used [25]. This allows the response of the material to variations in intensity to be separated from the influence of the pre-exposure. With the help of lateral scanning, the intensity response can be determined for each individual exposure and thus ultimately compared for different successively generated holograms, which differ in the amount of pre-exposure.

**Figure 4:**
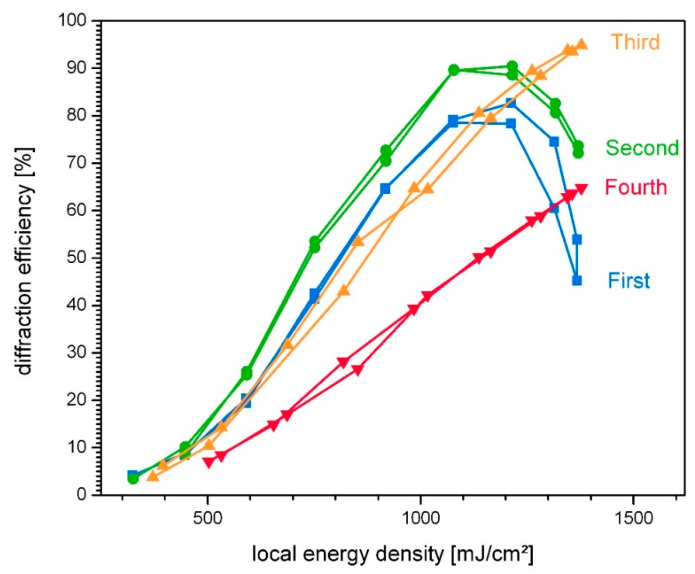
Impact of pre-exposure on the intensity response. All holograms are subsequently exposed (first, second, third, fourth) under equal conditions. Exposure duration was 15 s in each case.

Figure 4 shows lateral scans of a line of four subsequently exposed holograms, generated under equal conditions (laser power was constant 1.5 mW, exposure duration was 15 s in each case, and exposure geometry results in an unslanted grating with a period of Λ = 2.9 µm).

The lateral scans show the evolution of diffraction efficiency as a function of local energy density, which can be interpreted as intensity response. The intensity response of the four gratings differs significantly, confirming that the influence of pre-exposure due to pre-recorded holograms should not be underestimated. The lower sensitivity, resulting from consumption of photosensitive compounds on the one hand and prior crosslinking on the other hand, causes a decrease in the material response from exposure to exposure.

However, it must be emphasized that some degree of pre-exposure appears to have a stabilizing effect on regions exposed with low intensity: below 1200 mJ/cm^2^ the second grating shows the sharpest increase in the response curve (see green curve in Figure 4). While the first and second hologram are overmodulated (blue and green curve in Figure 4)—associated with a strong coupling and consequently high induced index contrast—the third and fourth grating (orange and red curve in Figure 4) show a progressively lower response, which is reflected in the low diffraction efficiency and, especially for the last grating, in a weak increase of the diffraction efficiency with increasing intensity.

#### 3.2.2. Interplay of Pre-Exposure and Energy Density of Exposure

**Figure 5:**
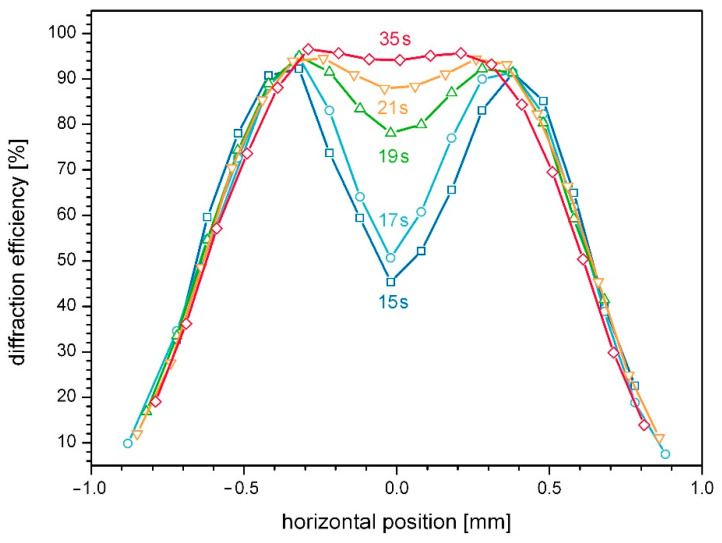
Interplay of exposure duration and pre-exposure on the intensity response. Gratings are subsequently created from short to long exposure duration, i.e., in the sequence 15 s (dark blue squares), 17 s (blue circles), 19 s (green triangles up), 21 s (orange triangles down) and 35 s (red diamonds).

To effectively estimate the impact of pre-exposure on the hologram performance, the interplay with the exposure dose—i.e., the product of exposure intensity and the exposure duration—is vitally important. Figure 5 shows lateral scans of a line of five subsequently exposed holograms, generated one after the other with increasing exposure duration.

For the lateral scans shown in Figure 5, the diffraction efficiency is displayed versus the lateral position, while in case of Figure 4, position was converted to local energy density. This way of presenting facilitates the spatial representation of the hologram shape. Superposition of pre-exposure influence and impact of exposure duration results in the case of the test series shown in Figure 5, in a clear preference of short exposure and a low pre-exposure level, while the overall outcome of the two effects, particularly in view of the very long exposure duration in the case of this example, is relatively moderate. Regardless of the similar impact of the respective effects, a potential mutual reinforcement is not to be expected. This gives rise to the assumption that a certain minimum material performance can be achieved reliably, regardless of the number of preceding exposures or for low power hologram recording in favor of longer exposure duration, respectively.

#### 3.2.3. Impact of Pre-Exposure on the Refractive Index Contrast

**Figure 6:**
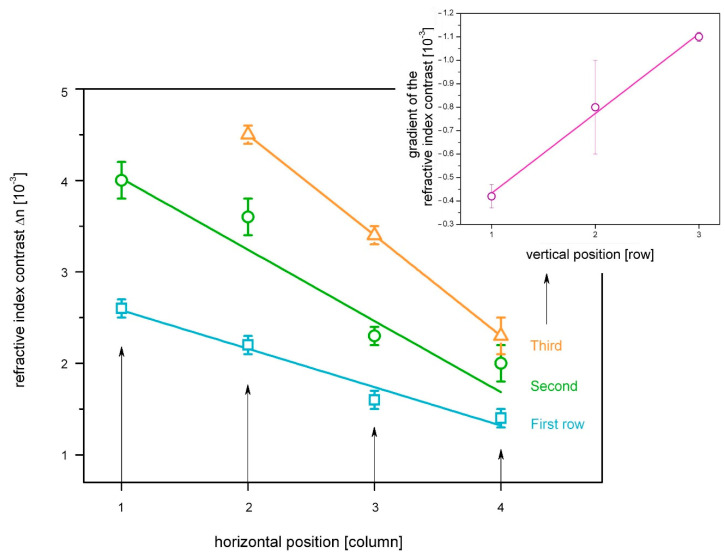
Impact of pre-exposure on the refractive index contrast. All holograms are subsequently exposed, line by line under equal conditions, i.e., in the first row (blue squares), second row (green circles), and the third row (orange triangles). Exposure duration was 15 s for each hologram. The inlay in the upper right shows the gradient of the refractive index contrast from line to line with corresponding linear fit.

The most important value characterizing a holographic material is the refractive index contrast Δ*n*, which is induced in the material by the holographic exposure. Therefore, it is particularly important here to determine the influence of the pre-exposure on the achievable contrast Δ*n*. For this purpose, three rows with four holograms each were generated and analyzed here in one sample. Results are shown in Figure 6, which illustrates the impact of pre-exposure by means of scattered light on the final refractive index contrast.

The results in Figure 6 clearly show that the refractive index contrast depends significantly on the pre-exposure of the sample. In fact, single exposures influence the value of the refractive index contrast by (30 ± 10)%. Within a single row, Δ*n* is decreasing from one column to the next. This effect is further enhanced by the total amount of pre-exposure, i.e., from line to line. It can be concluded that the refractive index contrast Δ*n* decreases with the number of previous exposures. This can be explained by gradual decrease of the photosensitivity as a result of photoacid and or monomer consumption. Similar phenomena, namely a reduced dynamic range with increasing pre-exposure dose, have been observed in the context of time-resolved studies, discussed in Section 3.1.

Another unexpected feature identifiable in Figure 6 concerns the vertical position of the holograms. Based on preliminary results, i.e., the pre-exposure influence (decrease of Δ*n*), a decrease is expected in Δ*n* from line to line. However, according to Figure 6, the vertical position on the sample seems to have a strong countervailing effect on the refractive index contrast, overcompensating for the pre-exposure influence. Holograms in line two and three are created subsequently and with a rising level of pre-exposure. Despite the significant amount of pre-exposure, the first grating in the third row exhibits the highest index contrast. Furthermore, the rise of Δ*n* from line to line shows a clear linear behavior (see inlay in Figure 6). To explain those effects, it must be considered that the samples have open surfaces, and a gradient of the layer thickness occurs due to a certain material flow. In addition, a monomer concentration gradient can be assumed as a result of the material flow. From the results in Figure 6 it can be seen that the influence of the material flow on the refractive index contrast exceeds that of the pre-exposure.

## 4. Conclusions

Based on investigations on a novel epoxy-based free surface volume holographic material, the influence of pre-exposure is studied, both on the formation of holograms in real-time as well as on their final, permanent diffraction characteristics. It could be shown that the influence of pre-exposure due to pre-recorded holograms should not be underestimated.

Results of real-time observation of holographic grating growth in doped and undoped SU-8 show the clear influence of pre-exposure on grating formation: the more pre-exposure the sample undergoes, the less strongly the grating growth rises and the later and slower diffusion starts and proceeds. This can be explained by a reduction of the dynamic range as a result of photoacid consumption, deceleration of the cross-linking, and exhaustion of non-cross-linked chains. The deceleration of diffusion can be explained by a certain loss in mobility of the matrix which impeded particle movement throughout prior cross-linking. Less easily explained is the fact that pre-exposed gratings in the doped material emerge with ever more complete null diffraction at the transition point.

Analysis of the intensity response has shown a decrease in the material response from exposure to exposure. Again, this can be explained by consumption of photosensitive compounds on the one hand and prior crosslinking on the other hand. However, it has been shown that some degree of pre-exposure appears to have a stabilizing effect on regions exposed with low intensity.

When analyzing the interaction of pre-exposure and exposure energy density, the best results were obtained with short exposure and a low pre-exposure level. It has been shown that the refractive index contrast Δ*n* decreases sharply with the level of pre-exposure. Again, both can be explained by decreased photosensitivity and prior crosslinking in the course of previous exposures.

In addition to refractive index contrast and energy density, another important parameter is the film thickness. The layer thickness represents a crucial factor for the selectivity of optical elements and for storage capacity of memories [26]. It remains to be clarified which role the pre-exposure plays here and also in connection with a variation of the exposure duration. In this context, the issue is volumetric shrinkage and expansion [25,27]. Here, too, the influence of the pre-exposure still has to be determined. Thus, it remains an exciting task to explore the mutual influence of material composition and processing, exposure, and pre-exposure conditions on grating formation in volume holographic materials.

## Data Availability

Not applicable.
